# *Bedtime Stories*: a sleep health education protocol for primary care clinicians, caregivers, and school-age children

**DOI:** 10.3389/frsle.2026.1787776

**Published:** 2026-05-07

**Authors:** Jessica M. Page, Grace Wang, Rebecca Robbins, Pallas Snider Ziporyn, Monica Ordway, Judith Owens

**Affiliations:** 1 Department of Psychiatry, Boston Children's Hospital, Boston, MA, United States; 2 Department of Neurology, Boston Children's Hospital, Boston, MA, United States; 3 Division of Sleep Medicine, Harvard Medical School, Boston, MA, United States; 4 Department of Medicine, Brigham and Women's Hospital, Boston, MA, United States; 5 Yale School of Nursing, Yale University, Orange, CT, United States

**Keywords:** caregiver, pediatric, provider, sleep health, socioecological approach

## Abstract

**Objectives:**

To examine the feasibility of *Bedtime Stories*, a sleep health education program designed to promote equitable pediatric sleep health among pediatric primary care clinicians (PCCs), caregivers, and school-aged children in community-based practice settings.

**Method:**

The *Bedtime Stories* program is informed by the socioecological model developed using the Consolidated Framework for Implementation Research, principles of social learning, and guidelines for feasibility pilot studies. The program includes three single-arm, pre–post pilot studies that will be conducted using: (1) a web-based provider sleep health education course (NCT06455579), (2) a digital caregiver sleep health intervention (NCT06618040), and (3) a children's book, *My Sleep Recipe*. The program's primary outcomes include benchmarks for participant recruitment and retention, along with metrics to evaluate feasibility and acceptability. Exploratory outcomes include providers' and caregivers' knowledge, self-efficacy, and changes in child sleep. Statistical analyses will focus on descriptive statistics and 95% confidence intervals.

**Results:**

Results will provide data on feasibility, engagement, and satisfaction for PCCs, caregivers, and school-aged children. Furthermore, they will provide preliminary insights into implementation barriers and facilitators for supporting sleep health practices, especially in underserved pediatric populations.

**Conclusions:**

This protocol outlines a scalable, multilevel framework for promoting pediatric sleep health. Findings will inform refinements and larger-scale trials to address sleep health practices through coordinated, equity-centered approaches that foster healthy sleep practices and reduce sleep health disparities across diverse communities.

## 1 Introduction

Sleep health is increasingly recognized as essential to physical and mental health, on par with nutrition and physical activity as key factors in disease prevention. Pediatric sleep health is a multidimensional construct composed of the following domains: behaviors (routines), satisfaction (sleep quality), alertness (ability to maintain wakefulness and vigilance during the day), timing (bedtimes and wake times), efficiency (continuity of sleep), and duration (total sleep time; B-SATED; Meltzer et al., 2021). In addition, the regularity of sleep (Buysse, 2014) schedules (i.e., lack of significant night-to-night variability and/or school night vs. “free” or non-school night variability, also termed social jet lag) may be equally important for sleep health (Beauvalet et al., 2017; Chen et al., 2022; Henderson et al., 2019). Deficits in these factors (e.g., insufficient sleep, poor sleep quality, frequent sleep interruptions, shifting sleep schedules) are associated with a myriad of adverse outcomes, including increased risk of obesity (Felso et al., 2017; Spruyt and Gozal, 2012), poor metabolic and cardiovascular health (Koren and Taveras, 2018), mood disorders, and cognitive dysfunction (Garrison, 2015; Goodlin-Jones et al., 2009; Miller et al., 2015; Saenz et al., 2015; Touchette et al., 2007; Wong et al., 2018) in children and adolescents.

Children living in socioeconomically stressed environments, as well as those in racially and ethnically marginalized communities, are at an even greater risk for sleep deficits, such as insufficient sleep and irregular sleep schedules (Ordway et al., 2023). Pediatric sleep health disparities are a burgeoning area of research, highlighting the impact of socioeconomic status, racism, discrimination, neighborhood segregation, social patterns, and access to health care (Billings et al., 2021) on sleep. Therefore, incorporating sleep health education in communities disproportionately experiencing poor sleep can foster healthy sleep practices early in life and potentially reduce these disparities (Uwah et al., 2025).

**Figure 1:**
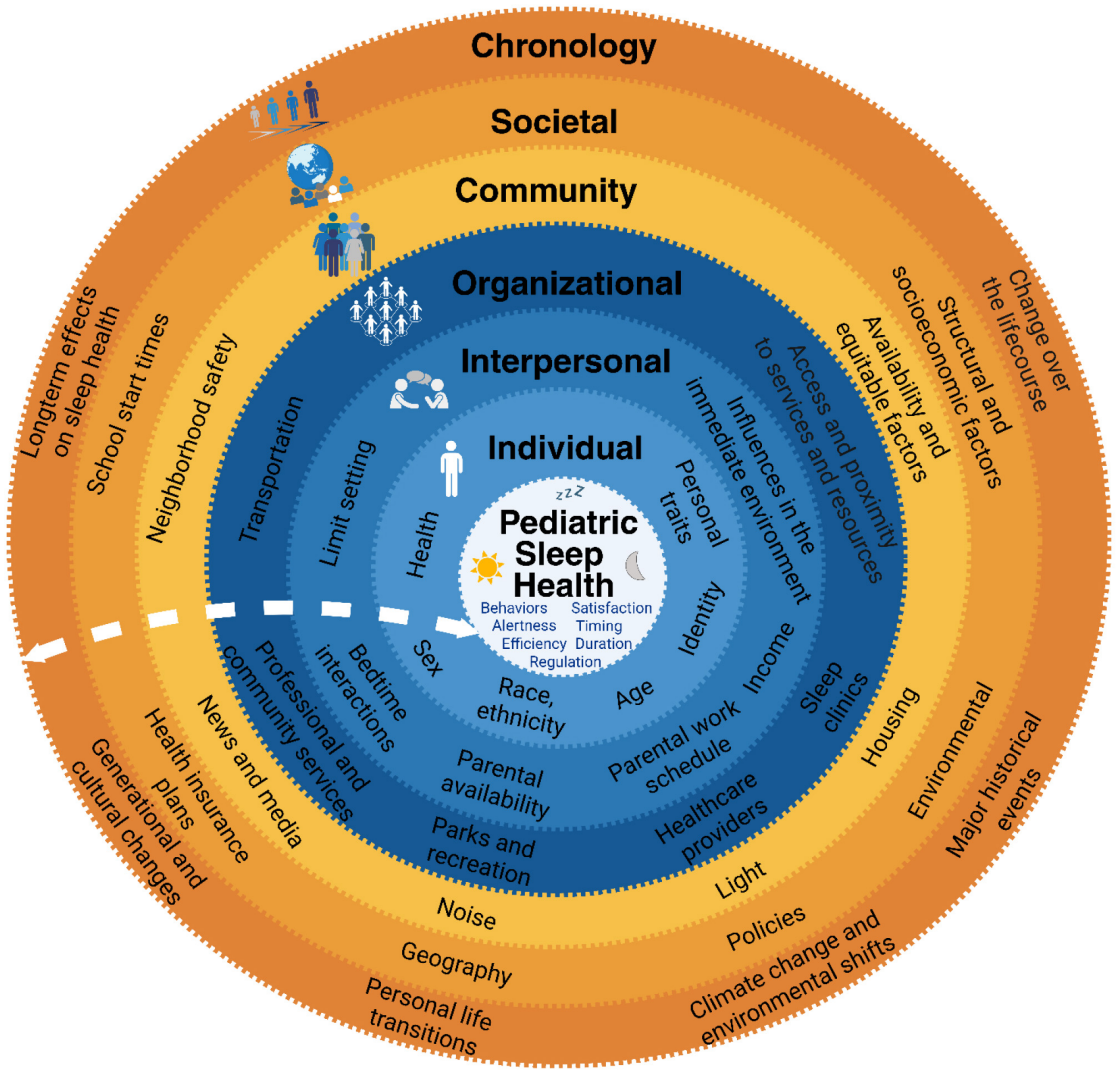
A socioecological approach to address sleep health disparities. This figure adapted with permission (Owens and Ordway, 2019) highlights the proximal and distal levels of the socioecological systems that contribute to sleep health practices, including disparities with comorbidities of sleep problems such as sleep disordered breathing in school-aged children.

Pediatric sleep is influenced by proximal and distal factors that can act as risk or protective influences, exacerbating or attenuating the relationship between sleep health and development. The Socioecological Model (Bronfenbrenner, 1994) provides a framework for targeting these influences on child sleep health and has been adapted (Uwah et al., 2025; Grandner, 2019; Blunden et al., 2023; Owens and Ordway, 2019; Redeker et al., 2018) to address sleep health disparities (Jackson et al., 2020). Figure 1 outlines an adapted socioecological model for understanding pediatric sleep health and sleep health disparities (Owens and Ordway, 2019). This model extends beyond the individual and focuses on modifiable socioecological factors to inform the development of feasible, effective, and equitable interventions at multiple levels. The model positions the individual (the child) at the center to emphasize the dynamic interplay among individual, interpersonal, organizational, community, and societal systems, as well as changes over time (the chronosystem), that shape development across the lifespan. Key levels of influence on sleep illustrated in Figure 1 include: interpersonal [e.g., relationships within the home that foster healthy sleep (Mindell et al., 2017, 2015)]; organizational [e.g., resources and supports within the pediatric primary care setting (Faruqui et al., 2011; Gruber et al., 2011) for sleep health education (Blunden and Rigney, 2015; Busch et al., 2017; Chen et al., 2023; Gruber et al., 2016)]; community (e.g., light Ohayon and Milesi, 2016; Paksarian et al., 2020; Tonon et al., 2022; Wang et al., 2022, noise Basner et al., 2023, and temperatures Basner et al., 2023; Berger et al., 2023 in the sleeping space); societal [e.g., structural racism, housing and education policies that contribute to poor sleep health (Attarian et al., 2024)]; and chronological [e.g., accumulation over time of sleep-disrupting factors (Jackson et al., 2020)].

The following *Bedtime Stories* protocol describes a comprehensive, three-pronged approach to developing sleep-health educational solutions to address identified gaps that contribute to sleep-health disparities in school-aged children. Sleep health education for pediatric primary care clinicians (PCCs) can promote routine surveillance of sleep practices in school-aged children (Owens and Ordway, 2019; Allen et al., 2016), support systematic screening to detect sleep-related risks or concerns (Blunden et al., 2023), and reinforce consistent messaging on the importance of healthy sleep. Increasing sleep health knowledge among PCCs may help improve the identification of sleep-related behaviors and contextual factors that contribute to poor sleep health. Despite the importance of early detection, screening, and anticipatory guidance for pediatric sleep health, practices within primary care settings remain inconsistent (Faruqui et al., 2011; Archbold et al., 2002; Owens, 2001; Honaker et al., 2018; Honaker and Meltzer, 2016; Meltzer et al., 2010), contributing to missed opportunities for prevention and intervention, particularly for school-aged children and adolescents (Honaker and Meltzer, 2016; Mindell et al., 1994, 2011). Primary care providers also serve as trusted sources of health information for families, reinforcing sleep as an essential health behavior. While face-to-face interactions during well-child visits are one important avenue for delivering sleep education, additional strategies (e.g., pre-visit surveys that include sleep questions, counseling by an affiliated nurse practitioner or behavioral health provider, and patient educational materials in the waiting room or online) may enhance dissemination. However, these approaches require PCCs to have a foundational understanding of sleep health and access to practical, user-friendly screening and other tools that will assist in recognizing and prioritizing sleep health implementation in clinical practice.

Sleep health education for caregivers is also critical, as caregivers are integral to and play a central role in shaping and providing oversight of their child's sleep health, family education is essential. However, most pediatric sleep health recommendations, including anticipatory guidance, have focused primarily on infants and young children (i.e., toddlers and preschoolers; Allam, 2024; Wilson et al., 2014), with comparatively little attention to school-aged children. Furthermore, while there are several school-based sleep health interventions targeting elementary, middle, and high school students (Blunden et al., 2023; Blunden and Rigney, 2015; Chen et al., 2023), these programs may insufficiently engage caregivers in their critical role of supervising and supporting children's sleep practices. In addition, there remains a dearth of programs designed for or evaluated within socioeconomically stressed or racially and ethnically marginalized communities. We sought to begin to address these gaps by focusing on sleep health practices that are school-age-specific, education methods that are family-centric and culturally sensitive, and by targeting caregivers while recognizing the increasing agency of older children in controlling their own sleep practices at a critical developmental stage.

Sleep health education for children is included as a key component of this protocol. Fostering healthy sleep habits in children and adolescents helps promote and sustain these behaviors into adulthood. For example, multiple studies have identified a positive, regular bedtime routine as an especially powerful means of facilitating sleep onset and increasing sleep duration (Mindell et al., 2017, 2015; Allen et al., 2016; Hale et al., 2011; Larsen and Jordan, 2022; Mindell and Williamson, 2018). Pre-sleep practices that include reading a bedtime book as a shared activity have the potential not only to provide an opportunity for introducing basics of sleep health in a non-didactic format, but also to potentially replace the use of electronic media at bedtime while promoting early literacy (Schlarb et al., 2025). However, many children's books feature imaginary, animal, or fictional characters rather than recognizable and relevant persons, often minimize or altogether avoid real-world sleep challenges (and solutions; Schlarb et al., 2020), and do not incorporate cultural content and considerations, which may influence a child's ability to engage with and internalize messages about sleep.

In summary, although prior interventions have employed a socioecological approach to address sleep health (Uwah et al., 2025; Grandner, 2019; Blunden et al., 2023; Redeker et al., 2018), none have simultaneously targeted multiple levels of influence, including PCCs, caregivers, and school-aged children. The overarching goal of the *Bedtime Stories* program is to improve children's sleep health and reduce sleep health disparities among primarily low-income, racially and ethnically diverse children and their families. We propose to develop and pilot-test a sleep health education program that incorporates a three-pronged approach targeting pediatric PCCs (*Bedtime Stories-Provider*), caregivers (*Bedtime Stories-Caregivers*), and children (*Bedtime Stories*-*Children*).

## 2 Methods

### 2.1 Theoretical frameworks

The socioecological framework directly informed the multilevel structure of the protocol and helps to ensure alignment of messaging across each level. We used the Consolidated Framework for Implementation Research (CFIR) constructs to inform planning, stakeholder engagement, and implementation processes. These frameworks also informed recruitment strategies, intervention adaptation, and tailoring and the evaluation of feasibility outcomes (Damschroder et al., 2009). We also integrated aspects of the Social Cognitive Theory (SCT; Bandura, 2001) to incorporate the important influences of on learning of cognitive, personal, and behavioral factors (e.g., skills, practice, self-efficacy) and environmental factors (e.g., social norms, access in the community, and influence on others to change the environment and adapt goals). Please see Table 1 for an overview of each framework and the strategies used to inform the development and implementation of this protocol.

**Table 1:** Integration of theoretical frameworks in the design of intervention.

Framework	Key constructs	Intervention level	Intervention component(s) informed
Socioecological	Cross-level	All levels	RESTED approach to support consistent, reinforcing sleep health messaging.
	Organizational level	Clinician	Practice guidance tools, workflow integration within primary care visits.
	Interpersonal level	Caregivers	Caregiver education materials, practical sleep routines, and strategies to support the sleep environment.
	Individual level	Child	Illustrated sleep health book targeting knowledge, routines, behavior, and development.
Social cognitive theory	Self-efficacy	Clinician, caregiver	Training materials, clinical pearls and measures to assess child sleep. Practical tools for implementing sleep routines.
	Outcome expectations	Clinician, caregiver	Education linking sleep and child health, behaviors, and academic outcomes.
	Observational learning	Caregiver, child	Characters modeling positive sleep behaviors, examples of structured routines and strategies for bedtime routines.
	Behavioral	Caregiver, child	Content supporting developmentally appropriate practices for sleep.
	Reinforcement	Caregiver, child	Encouraging consistency and the use of positive reinforcement strategies to support sleep.
Consolidated framework for implementation research	Intervention features (adaptability, complexity)	All levels	RESTED framework is integrated into routine well-child visits, weekly educational modules, and ingredients of a healthy sleep recipe.
	Inner setting (workflow, readiness for implementation)	Clinician	Identification of clinical champions, workflow tailoring for implementation.
	Outer setting (patient needs, community)	All levels	Equity and culturally focused adaptation of materials, and connection to sleep determinants.
	Characteristics of individuals (knowledge, clinician beliefs, self-efficacy)	Clinician	Clinician training to enhance knowledge and comfort with topics.
	Process (planning, engaging, executing, and evaluating)	Clinician	Implementation plan and discussions to support feasibility metrics and refining content.

#### 2.1.1 Development of the *bedtime stories* program

The *Bedtime Stories* program was developed by a team of clinical and scientific researchers and an advisory board of healthcare practitioners, caregivers, and community stakeholders (directors and PCCs from affiliated healthcare centers). By supporting and equipping clinicians with practical strategies and providing children with developmentally appropriate educational materials, the model aims to enhance alignment across levels that collectively shape sleep health practices. Although schools and other community settings (e.g., libraries) represent complementary pathways to disseminate information, particularly in communities that may experience barriers to healthcare access, these same communities would also benefit from increased and tailored sleep health promotion strategies in a healthcare setting, as they may be more vulnerable to the deleterious effects of poor sleep.

**Figure 2:**
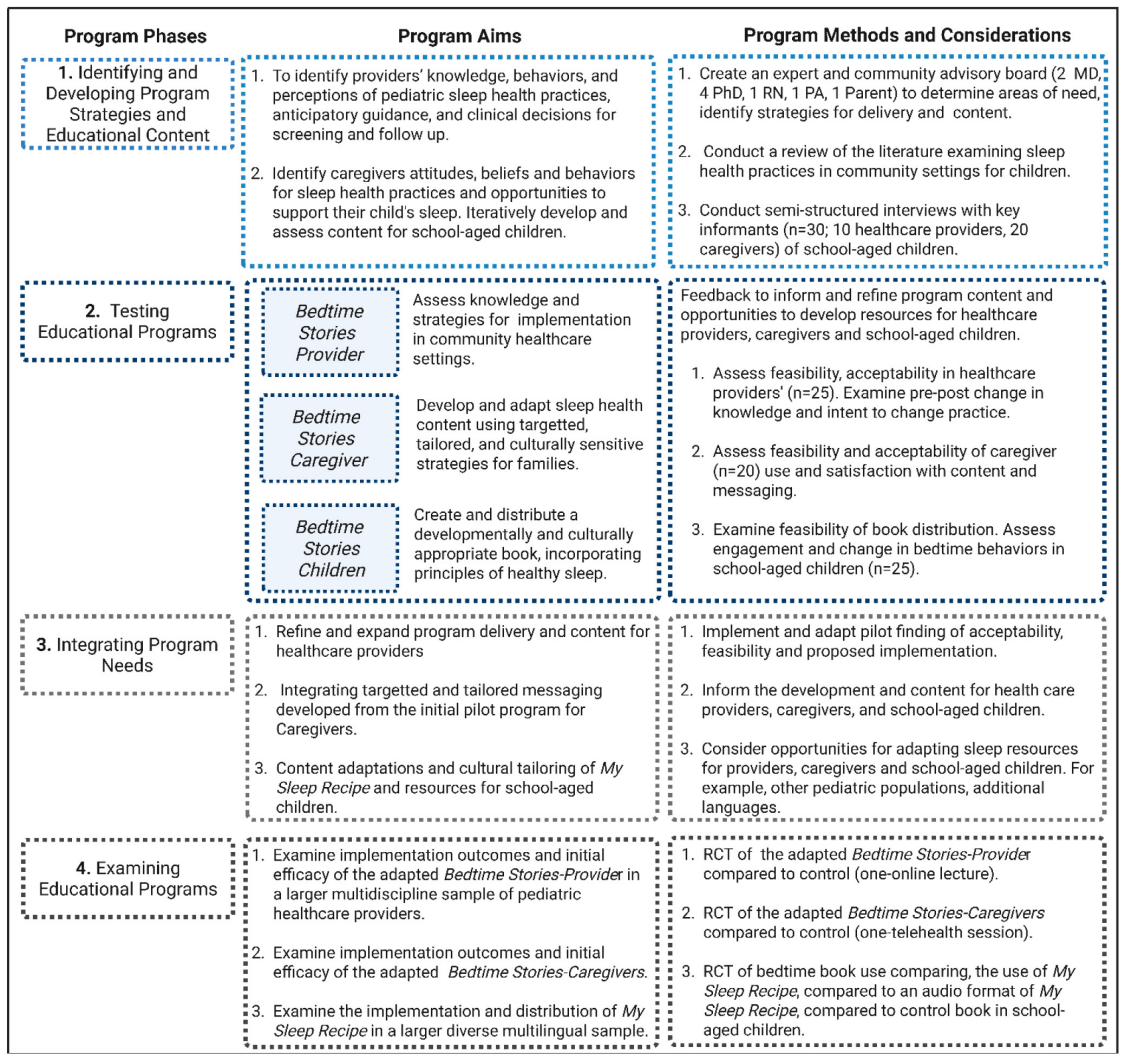
*Bedtime Stories* program development across each phase. Each phase describes the main activities and considerations for developing a multi-level study with three interventions with respective to specific aims, methods, and considerations for future adaptation and integration.

The advisory board met monthly throughout each phase of program development. Each phase of the *Bedtime Stories* program, along with plans for future adaptation and implementation, is outlined in Figure 2. Key milestones included: (1) delineation of the steps in the proposed development of the three sleep health interventions, including the collection of qualitative data from PCCs and caregivers to help inform content and message delivery; (2) identification of methodologies to assess the acceptability and feasibility of each intervention, including exploratory assessment of changes in sleep health knowledge and practices associated with the interventions (Moore et al., 2011; Teresi et al., 2022); (3) identification of opportunities for refining and adapting the educational components; and (4) development of strategies to support future adaptations and iterations for effectiveness trials.

#### 2.1.2 RESTED approach

To provide a unifying framework across these three educational components, we developed the “RESTED” acronym as an easily understandable approach to consistent sleep-health messaging for groups intended to help caregivers and providers understand and support healthy sleep practices. The RESTED (Routines, Expectations, Screen time, Timing, Environment, Duration) operationalizes and translates basic evidence-based sleep principles into a cohesive, developmentally adaptable form, creating a concise strategy for promoting pediatric sleep health through consistent, easily disseminated messaging. The RESTED approach builds upon established sleep hygiene principles by organizing evidence-based recommendations into an acronym format to promote clarity, consistency, and alignment of sleep health messaging across providers, caregivers, and children.

RESTED was developed by the senior author (blinded for review) based on a review of the literature as described earlier, including previous sleep health models such as B-SATED, and prior research (Mindell et al., 2015; Owens, 2001; Owens et al., 2000; Owens and Jones, 2011; Owens et al., 2011; Ersu et al., 2017). This integration is highlighted in Figure 2. For clinical care providers, the intervention includes in-depth content on sleep health for school-aged children, incorporating targeted components of the RESTED approach across multiple modules, but specifically highlighting these components in Modules 3 (Sleep Health) and 4 (Sleep Health Disparities). For caregivers, each weekly module is focused on a specific principle of the RESTED approach. For school-aged children, the book My Sleep Recipe incorporates key elements of the RESTED framework, while weaving this approach throughout to support healthy sleep practices. The following sections detail the study aims, procedures, and analytic plan of the *Bedtime Stories* program.

### 2.2 Phases of implementation

#### 2.2.1 Content development: review of literature

Educational content and proposed delivery mechanisms were informed by a scoping review of the literature examining sleep health practices in community settings for children to preadolescence (*manuscript in preparation*). We also conducted a systematic review of the literature examining sleep health practices in community settings and the impact on sleep duration in children and preadolescents (*manuscript in preparation*). From the literature search, we screened titles, abstracts, and full-text manuscripts that met our criteria. We extracted study samples, intervention details, and sleep health outcomes. The findings from both literature search strategies informed the development of educational content for stakeholder feedback.

#### 2.2.2 Content development: stakeholder feedback

We conducted semi-structured interviews with stakeholders to inform us about the delivery of intervention materials, implementation in designated settings (e.g., community healthcare centers, home), adaptations to content, and promotion strategies (Charmaz, 2006). We used qualitative methods to explore the perspectives of PCCs and caregivers of school-aged children on sleep health practices, factors that contribute to healthy sleep, and potential strategies to help mitigate potential risk and increase sleep promotion strategies. Specifically, we examined PCCs' current screening and follow-up practices during well-child visits, their perceptions regarding the importance of sleep for school-aged children, identified barriers to and facilitators of sleep health, and desired educational resources to address gaps for both practitioners and families. Caregivers of school-aged children were interviewed about their perspectives on the importance of sleep, factors that hinder or support healthy sleep, prior experiences accessing sleep-related information, and preferences for sleep health topics and desired resources. Details of the qualitative investigations, the analytical approach, and the findings from the interviews with providers (*under review)* and caregivers of school-aged children (*under review)* are presented in separate manuscripts.

### 2.3 *Bedtime Stories* program aims and procedures

#### 2.3.1 Aim 1. *Bedtime Stories*: providers sleep health education for PCCs

Table 2 summarizes the *Bedtime Stories* program aims and measures for each intervention. The primary aim is to assess the feasibility indicators (recruitment, retention, fidelity, adherence, engagement, and acceptability) of the *Bedtime Stories*: *Provider* online sleep health education course. An exploratory aim is to investigate changes in providers' knowledge and their intent to change clinical practice.

**Table 2:** Program aims.

Aim	Construct	Measure	Variable and method details
Aim 1. *Bedtime Stories-Provider* sleep health education for PCC			
1a	Feasibility	Feedback and evaluation questionnaire (FEQ)	Feasibility indicators (Teresi et al., 2022; recruitment, retention, fidelity, adherence, engagement, and acceptability). 19-item questionnaire-total score. Assessed once post course completion.
1b	Knowledge	Content knowledge	Content knowledge and clinical performance (CME/MOC questions via online modules). Knowledge is assessed at the end of every module. There are 4–6 quiz questions per module. To pass the course, one must receive a score of 80 or above for each of the required modules (6 modules).
		Topic knowledge and practices survey	Each topic is assessed for change in knowledge. 11-item questionnaire-pre-post change. Assessed once post course completion.
Aim 2. *Bedtime Stories-Caregiver* sleep health education for caregivers			
2a	Feasibility and acceptability	User timestamps, weekly check-ins	Feasibility will be assessed by examining engagement (number of families consented), retention (proportion of families who complete all modules). Acceptability of the *Bedtime Stories* program will be assessed weekly with a 4-item questionnaire during the weekly check-in. The digital questionnaire was developed specifically for the *Bedtime Stories* Caregiver intervention to assess acceptability, feasibility and utility of module content and resources.
2b	Caregiver sleep	Patient reported outcomes measurement information system (PROMIS): disturbances (Yu et al., 2012)	PROMIS sleep disturbances: 8-item questionnaire-total score. The adult PROMIS sleep disturbance instruments assess self-reported perceptions of sleep quality, sleep depth, and restoration associated with sleep. This includes perceived difficulties and concerns with getting to sleep or staying asleep, as well as perceptions of the adequacy of and satisfaction with sleep. The sleep disturbance short form assesses sleep disturbance over the past seven days using a 5-point Likert scale (1 = strongly disagree to 5 = strongly agree). Assessed at baseline and follow up.
		PROMIS sleep impairment (Yu et al., 2012)	PROMIS sleep impairment: 8-item questionnaire-total score. The adult PROMIS Sleep-Related Impairment examines self-reported perceptions of alertness, sleepiness, and tiredness during usual waking hours, and the perceived functional impairments during wakefulness associated with sleep problems or impaired alertness. The Sleep-Related Impairment short form assesses sleep-related impairment over the past seven days using a 5-point Likert scale (1 = strongly disagree to 5 = strongly agree). Assessed at baseline and follow up.
	Caregiver stress	Perceived Stress Scale (PSS; Cohen et al., 1983)	PSS: 10-item questionnaire; perceived stress-total score. The Perceived Stress Scale (PSS) is a measure of the degree to which situations in one's life are appraised as stressful. Items were designed to tap how unpredictable, uncontrollable, and overloaded respondents find their lives. Items from the PSS ask about feelings and thoughts during the last month using a 5-point Likert scale (0 = strongly disagree to 4 = strongly agree). Assessed at baseline.
	Caregiver knowledge and attitudes	Knowledge attitudes self-efficacy beliefs (KASB; Wilson et al., 2014) Questionnaire	KASB: 26-item questionnaire-total score. The KASB survey assesses parent knowledge, attitudes, self-efficacy, and beliefs. For each statement, respondents are asked to indicate how much you agree with each statement, on a 5-point Likert scale (0 = strongly disagree to 4 = strongly agree). Assessed at baseline and follow up.
	Child sleep	Child Sleep Environment: Children's Adolescents Sleep Environment Scale (CASES; Peltz et al., 2022)	CASES: 13-item parent report-total score. The CASES examines 3 domains: general environmental hazards (7 items), availability of bedding materials (2 items), and presence of electronics (4 items). The full scale and its subscales have shown strong discriminant reliability and generalizable across diverse demographic groups. Assessed at baseline.
		Sleep diary	Digital sleep diary: parent's completed 7-days of sleep diaries about their child's sleep. Parent report of-bedtime (average weekday and average weeknight), wake-up time, sleep onset, night awakenings (number and duration), sleep duration (average weekday and average weeknight), sleep quality. Assessed for 1-week at baseline and follow up.
		Patient reported outcomes measurement information system (PROMIS) pediatric sleep-disturbance (Forrest et al., 2018)	PROMIS pediatric and parent proxy sleep disturbance: 8-item parent report-total score. Assesses thoughts of one's sleep quality, and perceived difficulties with falling or staying asleep, sleep onset, and sleep continuity. The measures are universal rather than disease specific and assesses sleep-related impairment over the past 7 days using a 5-point Likert scale (1 = strongly disagree to 5 = strongly agree). Assessed at baseline and follow up.
		PROMIS pediatric sleep-related impairment (Forrest et al., 2018)	PROMIS pediatric and parent proxy sleep-related impairment: 8-item parent report-total score. The PROMIS pediatric and parent proxy sleep-related impairment item banks assess perceptions of sleepiness during usual awake hours and reported impairments during the day associated with sleep problems or daytime sleepiness. The measures are universal rather than disease specific and assesses sleep-related impairment over the past 7 days using a 5-point Likert scale (1 = strongly disagree to 5 = strongly agree). Assessed at baseline and follow up.
		Caregiver goals for child's sleep	Goals for children consist of 4-multiple choice, choose all that apply questions that were developed for this study. The questions examine the main reasons for wanting your child to get better sleep, main goals to improve your child's sleep, strategies to help achieve those goals and challenges to those goals. The goals for child sleep are assessed once at baseline.
Aim 3. *Bedtime Stories-Children* sleep health education for school-aged children			
3a	Feasibility	Brief bedtime book information questionnaire (BIQ)	BIQ: 11-item parent report. The BIQ was developed for this project. Caregiver's will provide the book number, healthcare center, and provider that distributed the book. There are 3 open-ended questions asking about cultural and content relevance. Assessed once after receiving the book.
3b	Engagement	Brief bedtime book information questionnaire (BIQ)	BIQ: 11-item parent report. The BIQ was developed for this project. There are 6 items used to assess general book use, bedtime routines, and book engagement. Items are rated using a 5-point Likert scale (1 = strongly disagree to 5 = strongly agree). Assessed once after receiving the book.

#### 2.3.2 Study design

A single-arm, pre–post feasibility study assessing feasibility, acceptability, and change in knowledge associated with a web-based sleep health education course for pediatric healthcare providers.

#### 2.3.3 Setting, recruitment, and eligibility criteria

Eligible participants (≥18 years of age) will be recruited from two community-based pediatric healthcare centers affiliated with a large academic medical center in the Northeast (*blinded for review*). Eligible participants are pediatric healthcare providers (MDs, RNs, PAs), including pediatric residents who conduct well-child visits for children aged 5–12 years, who will be recruited from two affiliate community health centers. Recruitment strategies will include institutional email and flyers with links to study information. To support recruitment, we have identified clinical champions, providers from each of the affiliated centers, who will help answer any questions about the research study. We will use verbal and clinical feedback from the clinical champion to identify barriers and solutions for recruitment and document reasons for non-participation. Those meeting the inclusion criteria and interested in participating will provide informed consent and complete a demographic form prior to study participation.

#### 2.3.4 Intervention description

**Figure 3:**
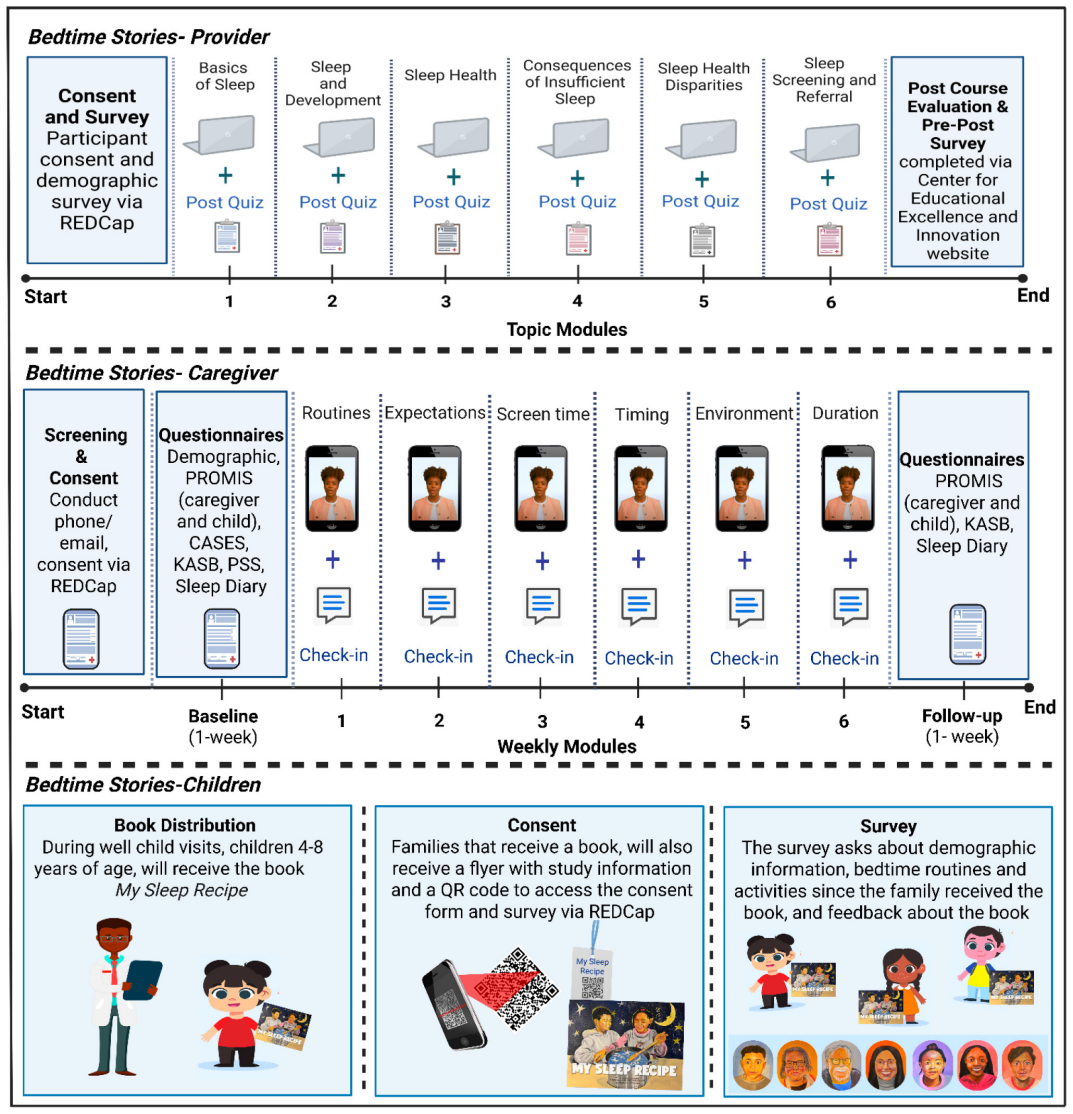
Design of the *Bedtime Stories* program and schematic of each intervention.

Figure 3 provides a schematic of the *Bedtime Stories* program. The *Bedtime Stories*-*Provider* course is designed to be a self-paced, interactive course hosted on the (*blinded for review*) Center for Educational Excellence and Innovation website. Participants will receive a personalized link to the *Bedtime Stories-Provider* course. The course includes six required modules (Basics of Sleep, Sleep and Child Development, Sleep Health, Consequences of Deficient Sleep, Sleep Health Disparities, Sleep Screening and Referral Procedures in Clinical Practice) and one optional module on Melatonin Use in Children. The course includes module learning objectives, module quiz questions, and downloadable resources. The course will take approximately 2 $\frac{1}{2}$−3 h to complete the six required modules. Content and topic knowledge will be assessed throughout and at the end of the course using a retrospective pretest–posttest survey. The retrospective pretest–posttest survey is a preferred method to reduce response shift (Visser et al., 2000) and uses a five-point Likert scale (1-strongly disagree; 5-strongly agree) to assess knowledge and intent to change practice. Participants will also complete a course feedback questionnaire and receive continuing medical education (CME) credit and a $25 gift card upon completion of the course.

#### 2.3.5 Outcome measures

We will assess the feasibility of implementing *Bedtime Stories-Provider* with participant recruitment (≥70%) and retention (retention ≥80% complete the course) targets. Adherence will be assessed by clinician completion rates. Fidelity will be assessed based on perceptions of the delivery of sleep guidance consistent with the RESTED framework during clinical encounters. Acceptability will be assessed via a post-course survey examining participants' satisfaction and relevance to clinical practice. Completion logs will be used to assess the participants' login frequency, time to complete the six required modules, and pass rate (the number of attempts to obtain 80% or more correct items per module quiz). Twenty-five participants completing all study milestones will be considered our target metric. Exploratory outcomes include pre–post change in topic knowledge across the six required modules, confidence in changing clinical practice, preferences for module topics, and satisfaction with course elements (e.g., videos, case studies, clinical pearls, text, quizzes, resources).

#### 2.3.6 Data analysis

Descriptive statistics (means, frequencies, percentages) will be used to summarize feasibility and acceptability. Feasibility will be examined by recruitment and retention (proportion of providers who complete the course). Exploratory paired pre-post comparisons will be presented with 95% confidence intervals (Tonon et al., 2022). We will investigate exploratory analyses to assess possible differences between healthcare professions (MD, NP, PA). Course evaluations and feedback will assess the acceptability and preferences of course topics and course elements to inform future adaptations and iterations.

#### 2.3.7 Ethical considerations

The *Bedtime Stories-Provider* study protocol is approved by the Institutional Review Board (P00044533) and registered with clinicaltrials.gov, NCT06455579 (version 2). Participants will provide informed consent electronically via RED Cap. Data confidentiality will be maintained in accordance with institutional standards. The results of this study will be presented at local, national, and international conferences and will be disseminated in peer-reviewed journals for publication.

### 2.4 Aim 2. *Bedtime Stories-Caregiver* sleep health education for caregivers

The primary aim will examine the feasibility of the 6-week *Bedtime Stories-Caregiver* program (recruitment, retention, fidelity, adherence, engagement, and acceptability). Planned exploratory investigations will examine changes in caregiver sleep, knowledge, and self-efficacy. Additional analyses will assess possible influences of baseline caregiver stress and the child's sleep environment on caregiver and child sleep at follow-up.

#### 2.4.1 Study design

A single-arm, pre–post feasibility pilot study of *Bedtime Stories-Caregiver*, a 6-week digital sleep health education intervention for caregivers of school-aged children.

#### 2.4.2 Setting, recruitment, and eligibility criteria

Caregivers will be recruited from two community health centers affiliated with a large hospital in the Northeast. Eligible participants are caregivers of children aged 5–12 years who receive care at the affiliated health centers, are comfortable communicating in English, and live in the same household as the child. Due to known variations in sleep physiology and sleep needs, exclusion criteria include: (1) children with a neurodevelopmental, neurological, genetic, or other diagnosis affecting neurodevelopment; (2) children with an identified or diagnosed sleep disorder; and/or (3) families who are currently homeless. Caregivers will be identified through electronic medical records and contacted via email or recruited in person at the healthcare center.

#### 2.4.3 Intervention description

As shown in the second row of Figure 3, once consented, caregivers will complete an onboarding survey about goals for their child's sleep (reasons for improving the child's sleep, goals, strategies, and anticipated challenges) and baseline questionnaires [PROMIS Sleep Disturbance and Sleep-Related Impairment short forms 8a, Perceived Stress Scale (PSS), and Knowledge, Attitudes, and Self-Efficacy Beliefs (KASB)] and for 1-week a daily digital sleep diary about their child's sleep. Beginning 1 week after baseline, for 6 weeks, caregivers will receive a weekly text message linking to a self-guided video module on one RESTED topic. In addition to the weekly video, participants will receive regularly scheduled reminders and check-ins. The video module reminders will be sent 3 days later if the video has not yet been viewed. Weekly check-ins will be sent 2 days after the video is viewed to promote engagement and gather feedback. Weekly check-ins will include brief questions about the child's sleep, perceived helpfulness of the content, and an opportunity for open-ended feedback for future adaptations. After 6 weeks, caregivers will complete follow-up questionnaires (via REDCap) and for 1 week, a daily digital sleep diary about their child's sleep. Participants will receive up to a $125 gift card upon completing study milestones (baseline, 6-weekly modules, follow-up).

#### 2.4.4 Outcome measures

Feasibility outcomes of interest will include recruitment (the number of caregivers consented), retention (proportion of participants who complete all modules), engagement, and acceptability. We will assess the feasibility of participant recruitment and retention with the planned enrolment of 30-40 participants. Our target for completing all modules and follow-up surveys (≥75%) with satisfaction ratings (≥80%) will be used as benchmark metrics. Fidelity of the intervention will be assessed with a question following the weekly content. We will evaluate adherence by having participants complete at least 4 of the 6 weekly modules and by using follow-up questionnaires (PROMIS Sleep Disturbance and Sleep-Related Impairment short forms (caregiver and child), the Knowledge, Attitudes, and Self-Efficacy Beliefs (KASB) Questionnaire, and sleep diary). A sample of 20 participants completing all study milestones (baseline, weekly modules, and follow-up) will be used as the benchmark target. We will monitor participant engagement with logged timestamps and assess acceptability using weekly satisfaction check-ins (2–3 days after viewing the weekly content) to assess the satisfaction and usefulness of the module content. As an exploratory aim, we will examine pre-post changes in caregiver sleep, knowledge, and self-efficacy, and child sleep.

#### 2.4.5 Data analysis

Planned analyses will focus on feasibility markers to inform future adaptations. Descriptive statistics, frequency, and percentages will be used to summarize the sample and to assess feasibility and acceptability. Child sleep data from the digital sleep diaries collected at baseline and follow-up will be used to assess sleep onset latency (time from initial lights-out until sleep onset), wake after sleep onset (time awake after initial sleep onset until last awakening), number of awakenings, total sleep time, sleep efficiency (total sleep time/time spent in bed × 100) and sleep quality rating (1-very poor to 5-excellent). Exploratory analyses will examine pre–post change in caregiver sleep (PROMIS-Sleep Disturbances, PROMIS-Sleep-Related Impairment), knowledge and self-efficacy (KASB), and changes in child sleep (PROMIS-Pediatric Short Form, Sleep Disturbances 8a, PROMIS-Pediatric Short Form, Sleep-Related Impairment 8a). The PSS for caregivers' stress and the CASES for the child's sleep environment will be used to examine possible influences of caregiver stress and the child's sleep environment on caregiver and child sleep at follow-up.

#### 2.4.6 Ethical considerations

*Bedtime Stories-Caregiver* is approved by the IRB (protocol P00047237) and registered with clinicaltrials.gov, NCT06618040 (version 2). Informed consent will be obtained from adult participants aged 18 years or older. Caregivers will provide informed electronic consent via REDCap. Participant confidentiality will be safeguarded by IRB requirements. Participation is voluntary, and participants can stop at any time at their discretion. The results of this study will be presented at local, national, and international conferences and will be disseminated in peer-reviewed journals for publication. Study resources and materials will be made available.

### 2.5 Aim 3. *Bedtime Stories-Children's* sleep health education for school-aged children

The primary aim is to investigate the feasibility of book distribution procedures, the acceptability of *My Sleep Recipes*, and to incorporate caregivers' comments and general feedback about the book into future iterations. As a secondary aim, we will assess child engagement with the book and changes in bedtime routines and sleep behaviors following the distribution of *My Sleep Recipe*.

#### 2.5.1 Study design

This is a single-arm pilot study examining the acceptability of and engagement with *My Sleep Recipe*. *My Sleep Recipe* was developed with input from the Bedtime Stories project community advisory board and incorporated qualitative data from caregiver interviews. The text was vetted through three sensitivity readers to ensure content validity for a culturally and linguistically diverse audience. *My Sleep Recipe* is a 32-page, illustrated softcover book for 4–8-year-old children, created with the expectation that caregivers will share the book at bedtime. The book features two racially, ethnically, and culturally diverse school-aged children who experience real-world sleep challenges and who generate practical solutions with the assistance of their families and healthcare providers to improve their sleep health and wellbeing. *My Sleep Recipe* uses the RESTED framework to convey basic principles of healthy sleep for school-aged children and includes suggestions for specific activities to reinforce sleep health messages.

#### 2.5.2 Setting, recruitment, and eligibility criteria

We will recruit participants receiving care from participating health centers within the Massachusetts League of Community Health Centers network and affiliated with a hospital network that has a Reach Out and Read program. We are looking to partner with healthcare centers that have a Reach Out and Read program, as these centers will have the experience and infrastructure already in place to support book distribution during well-child visits. Participant inclusion criteria include caregivers of children aged 4–8 years who reside in the same household, with English or Spanish proficiency.

Whereas the provider and caregiver materials are focused on supporting sleep health among children aged 5–12 years, the illustrated children's book My Sleep Recipe was intentionally designed for ages 4–8 years to help ensure developmental appropriateness and encourage mutually engaging, educational activity at bedtime for both the child and caregiver.

#### 2.5.3 Intervention description

We will distribute *My Sleep Recipe* to approximately 3–4 healthcare centers. Providers in these centers will distribute the *My Sleep Recipe* during well-child visits. Each book will contain a flyer with a QR code directing caregivers to REDCap for consent and a one-time survey capturing general demographics (caregiver's sex, age, race/ethnicity, etc.), feedback on *My Sleep Recipe*, perceptions about the book's content, perceived change in child's bedtime routines, and child sleep. The survey will take approximately 5–10 min to complete. Participants will receive a $20 gift card upon completing the survey.

#### 2.5.4 Outcome measures

Feasibility will be investigated by book distribution rates and survey completion rates. Recruitment feasibility will be measured through the distribution-to-response ratio. We will track the proportion of book recipients who complete a one-time survey. The target sample will be 25 participants who complete all study materials. Acceptability will be assessed by caregiver report of their child's sleep and satisfaction with the book's content (≥80% positive ratings). Book engagement will be measured as ≥3 book readings per week to be considered acceptable engagement. Other outcome measures will include the frequency of shared reading, changes in the bedtime routine, and the sleep environment (e.g., presence of electronic devices in the bedroom).

#### 2.5.5 Data analysis

Descriptive statistical analyses (means, frequencies, and percentages) and 95% confidence intervals will be used to summarize feasibility and acceptability metrics. Caregiver's feedback will inform future adaptations and recommendations. We will report descriptive statistics to examine changes in children's bedtime behaviors after receiving *My Sleep Recipe*. These data will be summarized as frequency and percentage counts.

#### 2.5.6 Ethical considerations

The *Bedtime Stories-Children* study protocol was approved by the Institutional Review Board (protocol P00045228). Caregivers will provide electronic informed consent and complete all surveys via REDCap. Participation is voluntary, and participants can stop at any time at their discretion. Findings from this study will be shared at local and national conferences and submitted for publication.

## 3 Discussion

The *Bedtime Stories* program is an innovative, multilevel intervention guided by the Socioecological framework (Bronfenbrenner, 1994), SCT (Bandura, 2001), and the CFIR models for implementation research, this protocol operationalizes these theories to address gaps in sleep health delivery within community-based primary care. Specifically, the Socioecological model informs the three levels (clinician, caregiver, and child). The SCT informs the behavioral change strategies embedded in the components of the RESTED approach. The CFIR model supports implementation planning and the feasibility of evaluation. By integrating and aligning these frameworks, this protocol was intentionally designed to examine the feasibility, acceptability, and implementation of delivering aligned sleep health messages across these levels. This alignment supports the research study aims of the respective interventions and serves as a program for reducing sleep health disparities (Billings et al., 2021; Blunden et al., 2023; Attarian et al., 2024).

### 3.1 Significance of *Bedtime Stories*

Each component of the *Bedtime Stories* program provides a unique, though complementary, contribution to support sleep health practices. *Bedtime Stories-Provider* focuses on improving PCCs knowledge and clinical practices in identifying and addressing sleep-related concerns by integrating evidence-based screening tools and anticipatory guidance into well-child visits (Blunden et al., 2023; Allam, 2024). This model has the potential to close educational gaps in pediatric training (Attarian et al., 2024; Allen et al., 2016; Honaker and Meltzer, 2016) and strengthen the clinical infrastructure for preventive sleep health practices. *Bedtime Stories-Caregiver* is designed to enhance caregiver awareness through practical, responsive strategies that support healthy sleep practices. Because caregivers play a central role in shaping their child's bedtime environment and routines, this study provides practical strategies for sleep health behaviors that are adaptable to families experiencing socioeconomic adversity, and also serves as a target to help address equity in sleep health education (Hiscock et al., 2019). *Bedtime Stories-Children* introduces a developmentally appropriate and culturally adapted bedtime book (*My Sleep Recipe*) that features families with diverse backgrounds and incorporates the real-world challenges that many children and families experience around sleep. This strategy is designed to empower children as active participants in their own sleep health, and even younger school-aged children, who are developing autonomy over their daily routines, can benefit from understanding the importance of regular sleep timing, duration, and quality. Embedding these lessons early on may help to foster lifelong health-promoting behaviors and reduce the risk for sleep-related problems (Blunden and Galland, 2014).

Collectively, these interventions represent a coordinated ecosystem that aligns healthcare, family, and education to improve sleep health equity. This multilevel alignment enhances opportunities for the reinforcement and sustainability of health practices. While previous research has demonstrated the efficacy of isolated sleep interventions (Williamson et al., 2022), few have integrated strategies across multiple ecological systems (Uwah et al., 2025; Redeker et al., 2018). The present model fills a critical gap by unifying healthcare and the family into a coherent program aimed at reducing sleep disparities experienced by children from racially and ethnically marginalized communities or those living in socioeconomically stressed environments. If effective, this approach could contribute to improved preventive care through earlier identification of sleep-related concerns and long-term benefits for children's health and development.

### 3.2 Anticipated barriers

Potential challenges include clinician time constraints within well-child visits, variability in workflow integration across practices, caregiver competing demands, and varying levels of support and access to resources. To mitigate these risks for clinical providers, we have included clinical champions in intervention design and implementation discussions to help address questions, and moreover, the protocol was intentionally designed to be brief, structured, and easily embedded within existing anticipatory guidance practices. We incorporate training modules and standardize messaging through the RESTED framework to reduce complexity and to support workflow alignment across practices. Feasibility metrics are also incorporated to detect early implementation barriers and to iteratively adapt and refine study components.

### 3.3 Contributions and future directions

We expect this work to contribute substantially to the fields of pediatric sleep health, and general pediatric health by broadly encompassing research, education, public health, and public policy initiatives related to the topic. Publications stemming from this project will serve to highlight the importance of pediatric sleep health as well as sleep health education, across the 3 target populations (clinicians, caregivers, and children) by providing a practical model of how to implement an ecological model with a sleep intervention framework that can be adapted to diverse community contexts.

Feasibility data will help optimize and guide implementation strategies prior to a fully powered efficacy trial. The multilevel design may serve as a model for integrating sleep health promotion into routine pediatric care, and lessons learned about aligning clinician, caregiver, and child messaging strategies may inform future research. Future research directions include testing effectiveness outcomes, incorporating direct child-level measures, expanding community reach efforts to the home or school setting, and examining long-term behavioral impact. We hope to encourage future studies to incorporate mixed method designs to expand beyond clinical and behavioral outcomes (e.g., sleep duration, quality, and timing) and to integrate contextual moderators such as caregiver stress, healthcare system capacity, and the environment (e.g., sleep space, neighborhood environment). In particular, longitudinal studies (Hale et al., 2011) are needed to examine sustained effects on sleep patterns and potential downstream effects on sleep and cardiometabolic health, and patient-reported outcomes such as emotional wellbeing and academic performance. Finally, given the use of artificial intelligence and the integration of sleep education into sleep health records, *Bedtime Stories* offers a model that can easily be integrated with digital health modalities, and future research should consider integrating digital health to harmonize intervention and data collection procedures.

### 3.4 Limitations and considerations for implementation

While we are proposing a tangible and feasible model, we acknowledge limitations that warrant further consideration. First, the efficacy and generalizability of *Bedtime Stories* interventions have not yet been established. This protocol represents a first step to addressing this limitation, as we will focus on feasibility indicators to inform future efficacy studies. There may be variability in program implementation across community healthcare sites, which may influence fidelity, participant recruitment, and outcomes. Given that *Betime Stories-Provider* and *Bedtime Stories-Caregiver* are both digital interventions, we hope this will address these potential limitations. We acknowledge that future scalable efforts will incorporate flexible delivery options (e.g., print-based materials, in-clinic distribution) to promote equitable access. Additionally, data collection may rely heavily on PCCs or caregiver reports of sleep behavior, which can be subject to bias. In the present protocol, the child-level engagement indicators in this feasibility pilot rely on caregiver-reported measures, which may not fully capture the child's experience and function as proxy assessments rather than direct measures of child behavioral change. Given the young age range (4–8 years) and the feasibility-focused design within community-based primary care settings, more direct child-centered assessment methods (e.g., observational approaches, brief semi-structured interviews) are beyond the scope of this initial phase. Future efficacy trials will incorporate direct child-level measures to more rigorously assess engagement, messaging fidelity, and behavioral change. Finally, given the current approach, continuous stakeholder engagement will be essential for maintaining program relevance and sustainability of implementation.

### 3.5 Conclusion

The *Bedtime Stories* program presents a novel socioecological framework for promoting equitable sleep health among school-aged children. Findings from its implementation will provide critical insights to inform future adaptation, dissemination, and policy integration of sleep health education programs.

## References

[B1] Allam A. (2024). Community Health Workers' Knowledge, Attitudes, Practices, and Awareness of American Academy of Pediatrics Recommendations of Safe Sleep Environments. Kent, OH: Kent State University.

[B2] Allen S. L. Howlett M. D. Coulombe J. A. Corkum P. V. (2016). ABCs of sleeping: a review of the evidence behind pediatric sleep practice recommendations. Sleep Med. Rev. 29, 1–14. doi: 10.1016/j.smrv.2015.08.006 26551999

[B3] Archbold K. H. Pituch K. J. Panahi P. Chervin R. D. (2002). Symptoms of sleep disturbances among children at two general pediatric clinics. J. Pediatr. 140, 97–102. doi: 10.1067/mpd.2002.119990 11815771

[B4] Attarian H. Dunietz G. L. Gavidia-Romero R. Jansen E. Johnson D. A. Kelman A. . (2024). Addressing sleep deserts: a proposed call for action. Sleep Health 10, S15–S18. doi: 10.1016/j.sleh.2023.09.008 37926658 PMC11181961

[B5] Bandura A. (2001). Social cognitive theory: an agentic perspective. Ann. Rev. Psychol. 52, 1–26. doi: 10.1146/annurev.psych.52.1.1 11148297

[B6] Basner M. Smith M. G. Jones C. W. Ecker A. J. Howard K. Schneller V. . (2023). Associations of bedroom PM2. 5, CO2, temperature, humidity, and noise with sleep: an observational actigraphy study. Sleep Health 9, 253–263. doi: 10.1016/j.sleh.2023.02.010 37076419 PMC10293115

[B7] Beauvalet J. C. Quiles C. L. Alves Braga de Oliveira M. Augusto Vieira Ilgenfritz C. Paz Loayza Hidalgo M. Tonon A. C. (2017). Social jetlag in health and behavioral research: a systematic review. ChronoPhysiol. Ther. 2017, 19–31. doi: 10.2147/CPT.S108750

[B8] Berger S. E. Ordway M. R. Schoneveld E. Lucchini M. Thakur S. Anders T. . (2023). The impact of extreme summer temperatures in the United Kingdom on infant sleep: implications for learning and development. Sci. Rep. 13:10061. doi: 10.1038/s41598-023-37111-2 37344536 PMC10284886

[B9] Billings M. E. Cohen R. T. Baldwin C. M. Johnson D. A. Palen B. N. Parthasarathy S. . (2021). Disparities in sleep health and potential intervention models: a focused review. Chest 159, 1232–1240. doi: 10.1016/j.chest.2020.09.249 33007324 PMC7525655

[B10] Blunden S. Galland B. (2014). The complexities of defining optimal sleep: empirical and theoretical considerations with a special emphasis on children. Sleep Med. Rev. 18, 371–378. doi: 10.1016/j.smrv.2014.01.002 24629828

[B11] Blunden S. McKellin W. Herdin T. Ipsiroglu O. S. (2023). Social-ecological considerations informing a universal screening strategy for sleep health in the community. Front. Psychiatry. 14:857717. doi: 10.3389/fpsyt.2023.857717 37020729 PMC10067715

[B12] Blunden S. Rigney G. (2015). Lessons learned from sleep education in schools: a review of dos and don'ts. J. Clin. Sleep Med. 11, 671–680. doi: 10.5664/jcsm.4782 25766709 PMC4442228

[B13] Bronfenbrenner U. (1994). Ecological models of human development. Int. Encyclopedia Educ. 3, 37–43.

[B14] Busch V. Altenburg T. M. Harmsen I. A. Chinapaw M. J. (2017). Interventions that stimulate healthy sleep in school-aged children: a systematic literature review. Euro. J. Public Health 27, 53–65. doi: 10.1093/eurpub/ckw140 28177474

[B15] Buysse D. J. (2014). Sleep health: can we define it? Does it matter? Sleep 37, 9–17. doi: 10.5665/sleep.3298 24470692 PMC3902880

[B16] Charmaz K. (2006). Measuring pursuits, marking self: meaning construction in chronic illness. Int. J. Qual. Stud. Health Wellbeing 1, 27–37. doi: 10.1080/17482620500534488

[B17] Chen C. X. Li T. M. H. Zhang J. Li S. X. Yu M. W. M. Tsang C. C. . (2022). The impact of sleep-corrected social jetlag on mental health, behavioral problems, and daytime sleepiness in adolescents. Sleep Med. 100, 494–500. doi: 10.1016/j.sleep.2022.09.027 36272246

[B18] Chen S. J. Li S. X. Zhang J. H. Lam S. P. Yu M. W. M. Tsang C. C. . (2023). School-based sleep education program for children: a cluster randomized controlled trial. MDPI 11:1853. doi: 10.3390/healthcare11131853 37444687 PMC10340294

[B19] Cohen S. Kamarck T. Mermelstein R. (1983). A global measure of perceived stress. J. Health Social Behav. 24, 385–396. doi: 10.2307/2136404 6668417

[B20] Damschroder L. J. Aron D. C. Keith R. E. Kirsh S. R. Alexander J. A. Lowery J. C. (2009). Fostering implementation of health services research findings into practice: a consolidated framework for advancing implementation science. Implement. Sci. 4:50. doi: 10.1186/1748-5908-4-50 19664226 PMC2736161

[B21] Ersu R. Boran P. Akin Y. Bozaykut A. Ay P. Yazar A. S. (2017). Effectiveness of a sleep education program for pediatricians. Pediatrics Int. 59, 280–285. doi: 10.1111/ped.13147 27566108

[B22] Faruqui F. Khubchandani J. Price J. H. Bolyard D. Reddy R. (2011). Sleep disorders in children: a national assessment of primary care pediatrician practices and perceptions. Pediatrics 128, 539–546. doi: 10.1542/peds.2011-0344 21873695

[B23] Felso R. Lohner S. Hollódy K. Erhardt É. Molnár D. (2017). Relationship between sleep duration and childhood obesity: systematic review including the potential underlying mechanisms. Nutr. Metab. Cardiovasc. Dis. 27, 751–761. doi: 10.1016/j.numecd.2017.07.008 28818457

[B24] Forrest C. B. Meltzer L. J. Marcus C. L. de la Motte A. Kratchman A. Buysse D. J. . (2018). Development and validation of the PROMIS pediatric sleep disturbance and sleep-related impairment item banks. Sleep 41:zsy054. doi: 10.1093/sleep/zsy054 29546286

[B25] Garrison M. M. (2015). The feedback whirlpool of early childhood sleep and behavior problems. JAMA Pediatr. 169, 525–526. doi: 10.1001/jamapediatrics.2015.0356 25868054

[B26] Goodlin-Jones B. Tang K. Liu J. Anders T. F. (2009). Sleep problems, sleepiness and daytime behavior in preschool-age children. J. Child Psychol. Psychiatry 50, 1532–1540. doi: 10.1111/j.1469-7610.2009.02110.x 19573036

[B27] Grandner M. A. (2019). “Social-ecological model of sleep health,” in Sleep and Health, ed. M. A. Grandner (London: Elsevier), 45–53. doi: 10.1016/B978-0-12-815373-4.00005-8

[B28] Gruber R. Cassoff J. Knäuper B. (2011). Sleep health education in pediatric community settings: rationale and practical suggestions for incorporating healthy sleep education into pediatric practice. Pediatric Clin. 58, 735–754. doi: 10.1016/j.pcl.2011.03.006 21600352

[B29] Gruber R. Somerville G. Bergmame L. Fontil L. Paquin S. (2016). School-based sleep education program improves sleep and academic performance of school-age children. Sleep Med. 21, 93–100. doi: 10.1016/j.sleep.2016.01.012 27448478

[B30] Hale L. Berger L. M. LeBourgeois M. K. Brooks-Gunn J. (2011). A longitudinal study of preschoolers' language-based bedtime routines, sleep duration, and well-being. J. Fam. Psychol. 25:423. doi: 10.1037/a0023564 21517173 PMC3134391

[B31] Henderson S. E. Brady E. M. Robertson N. (2019). Associations between social jetlag and mental health in young people: a systematic review. Chronobiol. Int. 36, 1316–1333. doi: 10.1080/07420528.2019.1636813 31387413

[B32] Hiscock H. Quach J. Paton K. Peat R. Gold L. Arnup S. . (2019). Impact of a behavioral sleep intervention on new school entrants' social emotional functioning and sleep: a translational randomized trial. Behav. Sleep Med. doi: 10.1080/15402002.2018.1469493 29757013

[B33] Honaker S. M. Dugan T. Daftary A. Davis S. Saha C. Baye F. . (2018). Unexplained practice variation in primary care providers' concern for pediatric obstructive sleep apnea. Acad. Pediatr. 18, 418–424. doi: 10.1016/j.acap.2018.01.011 29391284

[B34] Honaker S. M. Meltzer L. J. (2016). Sleep in pediatric primary care: a review of the literature. Sleep Med. Rev. 25, 31–39. doi: 10.1016/j.smrv.2015.01.004 26163054

[B35] Jackson C. L. Walker J. R. Brown M. K. Das R. Jones N. L. (2020). A workshop report on the causes and consequences of sleep health disparities. Sleep 43:zsaa037. doi: 10.1093/sleep/zsaa037 32154560 PMC7420527

[B36] Koren D. Taveras E. M. (2018). Association of sleep disturbances with obesity, insulin resistance and the metabolic syndrome. Metab. Clin. Exp. 84, 67–75. doi: 10.1016/j.metabol.2018.04.001 29630921

[B37] Larsen K. L. Jordan S. S. (2022). Factors associated with consistent bedtime routines and good sleep outcomes. Children's Health Care 51, 139–162. doi: 10.1080/02739615.2021.1981331

[B38] Meltzer L. J. Johnson C. Crosette J. Ramos M. Mindell J. A. (2010). Prevalence of diagnosed sleep disorders in pediatric primary care practices. Pediatrics 125, e1410–e1418. doi: 10.1542/peds.2009-2725 20457689 PMC3089951

[B39] Meltzer L. J. Williamson A. A. Mindell J. A. (2021). Pediatric sleep health: it matters, and so does how we define it. Sleep Med. Rev. 57:101425. doi: 10.1016/j.smrv.2021.101425 33601324 PMC9067252

[B40] Miller A. L. Seifer R. Crossin R. Lebourgeois M. K. (2015). Toddler's self-regulation strategies in a challenge context are nap-dependent. J. Sleep Res. 24, 279–287. doi: 10.1111/jsr.12260 25394169 PMC4430484

[B41] Mindell J. A. Bartle A. Wahab N. A. Ahn Y. Ramamurthy M. B. Huong H. T. D. . (2011). Sleep education in medical school curriculum: a glimpse across countries. Sleep Med. 12, 928–931. doi: 10.1016/j.sleep.2011.07.001 21924951

[B42] Mindell J. A. Leichman E. S. Lee C. Williamson A. A. Walters R. M. (2017). Implementation of a nightly bedtime routine: how quickly do things improve? Infant Behav. Dev. 49, 220–227. doi: 10.1016/j.infbeh.2017.09.013 28985580 PMC6587179

[B43] Mindell J. A. Li A. M. Sadeh A. Kwon R. Goh D. Y. (2015). Bedtime routines for young children: a dose-dependent association with sleep outcomes. Sleep 38, 717–722. doi: 10.5665/sleep.4662 25325483 PMC4402657

[B44] Mindell J. A. Moline M. L. Zendell S. M. Brown L. W. Fry J. M. (1994). Pediatricians and sleep disorders: training and practice. Pediatrics 94, 194–200. doi: 10.1542/peds.94.2.194 8036073

[B45] Mindell J. A. Williamson A. A. (2018). Benefits of a bedtime routine in young children: sleep, development, and beyond. Sleep Med. Rev. 40, 93–108. doi: 10.1016/j.smrv.2017.10.007 29195725 PMC6587181

[B46] Moore C. G. Carter R. E. Nietert P. J. Stewart P. W. (2011). Recommendations for planning pilot studies in clinical and translational research. Clin. Transl. Sci. 4, 332–337. doi: 10.1111/j.1752-8062.2011.00347.x 22029804 PMC3203750

[B47] Ohayon M. M. Milesi C. (2016). Artificial outdoor nighttime lights associate with altered sleep behavior in the American general population. Sleep 39, 1311–1320. doi: 10.5665/sleep.5860 27091523 PMC4863221

[B48] Ordway M. R. Sadler L. S. Jeon S. Pierre J. C. Canapari C. Redeker N. S. (2023). Early emergence of racial and ethnic differences in sleep health among toddlers living in low-income families. Sleep Health 9, 389–397. doi: 10.1016/j.sleh.2023.02.006 37453903 PMC10517059

[B49] Owens J. Ordway M. (2019). “Sleep among children,” in The Social Epidemiology of Sleep, ed. M. A. Grandner (Oxford: Oxford University Press), 93–120. doi: 10.1093/oso/9780190930448.003.0004

[B50] Owens J. Spirito A. McGuinn M. (2000). Children's Sleep Habits Questionnaire (CSHQ). Washington, DC: American Psychological Association. doi: 10.1037/t33022-000 11145319

[B51] Owens J. A. (2001). The practice of pediatric sleep medicine: results of a community survey. Pediatrics 108:e51. doi: 10.1542/peds.108.3.e51 11533369

[B52] Owens J. A. Jones C. (2011). Parental knowledge of healthy sleep in young children: results of a primary care clinic survey. J. Dev. Behav. Pediatrics 32, 447–453. doi: 10.1097/DBP.0b013e31821bd20b 21546852

[B53] Owens J. A. Jones C. Nash R. (2011). Caregivers' knowledge, behavior, and attitudes regarding healthy sleep in young children. J. Clin. Sleep Med. 7, 345–350. doi: 10.5664/JCSM.1186 21897770 PMC3161766

[B54] Paksarian D. Rudolph K. E. Stapp E. K. Dunster G. P. He J. Mennitt D. . (2020). Association of outdoor artificial light at night with mental disorders and sleep patterns among US adolescents. JAMA Psychiatry 77, 1266–1275. doi: 10.1001/jamapsychiatry.2020.1935 32639562 PMC7344797

[B55] Peltz J. S. Rogge R. D. Elmore-Staton L. Spilsbury J. Buckhalt J. A. (2022). The development of a scale to assess children's and adolescents' sleep environments. J. Clin. Sleep Med. 18, 2353–2365. doi: 10.5664/jcsm.10110 35702021 PMC9516582

[B56] Redeker N. S. Ordway M. R. Banasiak N. Caldwell B. Canapari C. Crowley A. . (2018). Community partnership for healthy sleep: research protocol. Res. Nurs. Health 41, 19–29. doi: 10.1002/nur.21840 29277901 PMC5780228

[B57] Saenz J. Yaugher A. Alexander G. M. (2015). Sleep in infancy predicts gender specific social-emotional problems in toddlers. Front. Pediatrics 3:42. doi: 10.3389/fped.2015.00042 26029685 PMC4426713

[B58] Schlarb A. A. Blunden S. Brand S. Bruni O. Corkum P. Horne R. S. C. . (2025). The future of paediatric sleep medicine: a blueprint for advancing the field. J. Sleep Res. 34:e14482. doi: 10.1111/jsr.14482 40151923 PMC12426711

[B59] Schlarb A. A. Kater M. J. Werner A. Landwehr J. Kolip P. Cattarius B. . (2020). When the sleep fairy's magic does not work—a critical analysis of children's book about sleep. Somnologie 24, 229–236. doi: 10.1007/s11818-020-00278-1

[B60] Spruyt K. Gozal D. (2012). The underlying interactome of childhood obesity: the potential role of sleep. Childhood Obesity 8, 38–42. doi: 10.1089/chi.2011.0105 22799478 PMC3647589

[B61] Teresi J. A. Yu X. Stewart A. L. Hays R. D. (2022). Guidelines for designing and evaluating feasibility pilot studies. Med. Care 60, 95–103. doi: 10.1097/MLR.0000000000001664 34812790 PMC8849521

[B62] Tonon A. C. Constantino D. B. Amando G. R. Abreu A. C. Francisco A. P. de Oliveira M. A. B. . (2022). Sleep disturbances, circadian activity, and nocturnal light exposure characterize high risk for and current depression in adolescence. Sleep 45:zsac104. doi: 10.1093/sleep/zsac104 35522984

[B63] Touchette E. Petit D. Séguin J. R. Boivin M. Tremblay R. E. Montplaisir J. Y. (2007). Associations between sleep duration patterns and behavioral/cognitive functioning at school entry. Sleep 30, 1213–1219. doi: 10.1093/sleep/30.9.1213 17910393 PMC1978413

[B64] Uwah E. A. Cicalese O. Davis B. Neelapu M. Steinberg G. Handa A. . (2025). Socioecological factors linked to co-occurring early childhood sleep health disparities and developmental outcomes: protocol for the sleep in preschoolers cross-sectional study. BMJ Open 15:e100956. doi: 10.1136/bmjopen-2025-100956 40118487 PMC11931971

[B65] Visser M. R. Smets E. M. Sprangers M. A. de Haes H. J. (2000). How response shift may affect the measurement of change in fatigue. J. Pain Symptom Manage. 20, 12–18. doi: 10.1016/S0885-3924(00)00148-2 10946164

[B66] Wang L. B. Gong Y. C. Fang Q. L. Cui X. X. Dharmage S. C. Jalaludin B. . (2022). Association between exposure to outdoor artificial light at night and sleep disorders among children in China. JAMA Network Open 5:e2213247. doi: 10.1001/jamanetworkopen.2022.13247 35594042 PMC9123501

[B67] Williamson A. A. Okoroji C. Cicalese O. Evans B. C. Ayala A. Harvey B. . (2022). Sleep Well! An adapted behavioral sleep intervention implemented in urban primary care. J. Clin. Sleep Med. 18, 1153–1166. doi: 10.5664/jcsm.9822 34910624 PMC8974371

[B68] Wilson K. E. Miller A. L. Bonuck K. Lumeng J. C. Chervin R. D. (2014). Evaluation of a sleep education program for low-income preschool children and their families. Sleep 37, 1117–1125. doi: 10.5665/sleep.3774 24882907 PMC4015386

[B69] Wong M. M. Puttler L. I. Nigg J. T. Zucker R. A. (2018). Sleep and behavioral control in earlier life predicted resilience in young adulthood: a prospective study of children of alcoholics and controls. Addict. Behav. 82, 65–71. doi: 10.1016/j.addbeh.2018.02.006 29494860 PMC5880316

[B70] Yu L. Buysse D. J. Germain A. Moul D. E. Stover A. Dodds N. E. . (2012). Development of short forms from the PROMIS™ sleep disturbance and sleep-related impairment item banks. Behav. Sleep Med. 10, 6–24. doi: 10.1080/15402002.2012.636266 22250775 PMC3261577

